# Evaluating a customised large language model (DELSTAR) and its ability to address medication-related questions associated with delirium: a quantitative exploratory study

**DOI:** 10.1007/s11096-025-01900-8

**Published:** 2025-04-10

**Authors:** Katharina Teresa Spagl, Edward William Watson, Adam Jatowt, Anita Elaine Weidmann

**Affiliations:** 1 Department of Clinical Pharmacy, Institute of Pharmacy, Innsbruck University, Innrain 80, 6020 Innsbruck, Austria; 2 Department of Media and Learning Technology, Innsbruck University, Innrain 52, 6020 Innsbruck, Austria; 3 Department of Computer Science and Digital Science Centre, Innsbruck University, Technikerstraße 21a, 6020 Innsbruck, Austria

**Keywords:** Clinical pharmacy information systems, Delirium, Drug prescribing, Intelligence artificial, Intelligence machine, Patient safety

## Abstract

**Background:**

A customised large language model (LLM) could serve as a next-generation clinical pharmacy research assistant to prevent medication-associated delirium. Comprehensive evaluation strategies are still missing.

**Aim:**

This quantitative exploratory study aimed to develop an approach to comprehensively assess the domain-specific customised delirium LLM (DELSTAR) ability, quality and performance to accurately address complex clinical and practice research questions on delirium that typically require extensive literature searches and meta-analyses.

**Method:**

DELSTAR, focused on delirium-associated medications, was implemented as a ‘Custom GPT’ for quality assessment and as a Python-based software pipeline for performance testing on closed and leading open models. Quality metrics included statement accuracy and data credibility; performance metrics covered F1-Score, sensitivity/specificity, precision, AUC, and AUC-ROC curves.

**Results:**

DELSTAR demonstrated more accurate and comprehensive information compared to information retrieved by traditional systematic literature reviews (SLRs) (*p* < 0.05) and accessed Application Programmer Interfaces (API), private databases, and high-quality sources despite mainly relying on less reliable internet sources. GPT-3.5 and GPT-4o emerged as the most reliable foundation models. In Dataset 2, GPT-4o (F1-Score: 0.687) and Llama3-70b (F1-Score: 0.655) performed best, while in Dataset 3, GPT-3.5 (F1-Score: 0.708) and GPT-4o (F1-Score: 0.665) led. None consistently met desired threshold values across all metrics.

**Conclusion:**

DELSTAR demonstrated potential as a clinical pharmacy research assistant, surpassing traditional SLRs in quality. Improvements are needed in high-quality data use, citation, and performance optimisation. GPT-4o, GPT-3.5, and Llama3-70b were the most suitable foundation models, but fine-tuning DELSTAR is essential to enhance sensitivity, especially critical in pharmaceutical contexts.

**Supplementary Information:**

The online version contains supplementary material available at 10.1007/s11096-025-01900-8.

## Impact statements

- A delirium-specific large language model (DELSTAR) that focuses on medication-related information was developed.
- This customised, domain-specific chatbot using a simplified tree-of-thoughts version serves as a proof-of-concept to support future literature-based clinical practice research.
- DELSTAR’s ability to identify accurate and comprehensive information about medication-related causes and treatments of delirium could be beneficial to support clinical prescribing practice in the future.

## Introduction

Delirium, an acute non-specific neuropsychiatric syndrome, remains a major clinical challenge, increasing morbidity, mortality and the risk of long-term cognitive decline [1–5]. Despite its significant impact on patient’s health, the pathophysiology remains unclear, but it likely arises from multiple factors, including medication [4, 6, 7]. This acute yet debilitating condition primarily affects vulnerable groups, such as the elderly, the critically ill, and patients in the peri-operative care setting [5, 8–10]. Advancements in medical technologies, particularly in deep medicine (DM), have shown that AI-driven tools can enhance patient diagnosis and treatment by processing and analysing clinical data [11]. However, AI’s applications in clinical contexts require thorough validation due to patient safety risks [12]. While interest in AI tools is growing, understanding of their mechanisms and appropriate use remains limited [12, 13].

Despite drug toxicity being estimated to account for 12–39% of all delirium cases, comprehensive documentation on medication-associated risks and related information remains lacking [14]. While several guidelines recommend that all patients at risk of delirium should have a medication review conducted by a clinical pharmacist, detailed prescribing guidance is not available [15, 16]. To address this gap, the Institute of Clinical Pharmacy at the University of Innsbruck aims to create comprehensive documentation on medication-associated delirium in diverse patient populations (e.g. dementia, paediatrics, peri-operative). Complementing several high-quality systematic literature reviews (SLR) on medication risks associated with delirium in these populations [17–24], the team developed DELSTAR, a customised, domain-specific large language model (LLM), as a novel research assistant [25]. DELSTAR, unlike existing delirium specific predictive models that do not include medication as a predictive variable, focuses specifically on medication related information and causes [25]. By targeting collections of peer reviewed papers as a source data, using application program interfaces (APIs) where possible, the DELSTAR AI tool embodies a novel approach for clinical pharmacy research [25]*.* In natural language processing (NLP), LLMs like DELSTAR are prone to “hallucinations,” where they may produce incorrect but confident answers, raising concerns about transparency and accountability [26, 27]. To ensure the safe and effective integration of LLMs in clinical and research settings, it is essential to establish precise evaluation methods for assessing their quality and performance [11, 12]. While general benchmarks like GLUE, SuperGLUE, and BIG-Bench assess LLM capabilities, and frameworks like AdvGLUE and TextFlint test performance and robustness [28–30], no comprehensive evaluation methods exist for customised LLMs in clinical pharmacy applications like DELSTAR. This study aims to address these evaluation challenges by testing statement quality, data integration, credibility and performance testing.

### Aim

This quantitative exploratory study aimed to develop an approach to comprehensively assess the domain-specific customised delirium LLM’s (DELSTAR) ability, quality and performance to accurately address complex research questions on medication-associated delirium that typically require extensive literature searches and meta-analyses.

### Ethics approval

No ethical approval was required for this machine learning evaluation study. The guidelines of the Austrian Agency for Research Integrity on Good Scientific Practice were adhered to [31].

## Method

### DELSTAR development

DELSTAR’s core consists of its dataset and system prompt, which are compatible with various backend or foundation models. Iterative updates improved response quality. Notably, DELSTAR was designed so that no external dependencies on cloud systems arise. Two implementations were developed: firstly, a “Custom GPT” using OpenAI’s web interface for quality testing; secondly, a locally run Python pipeline using LLamaIndex to test performance at a larger scale. The Python version supports OpenAI’s closed and open models like Llama and Mistral, enabling large-scale automated inferencing and data extraction. The LLM user interface tool ‘Open Web UI’ was chosen as the external tool, since it offers a user-friendly frontend interface similar to the OpenAI web interface. This choice facilitated a more direct comparison between the open and closed foundation models capable of driving DELSTAR (V4 and V5). Challenges in restricting the pipeline outputs to “yes” or “no” were resolved using a “Yes–no-summary-bot,” built on Llama3.1, to make binary judgments.

### DELSTAR training

**Fig. 1:**
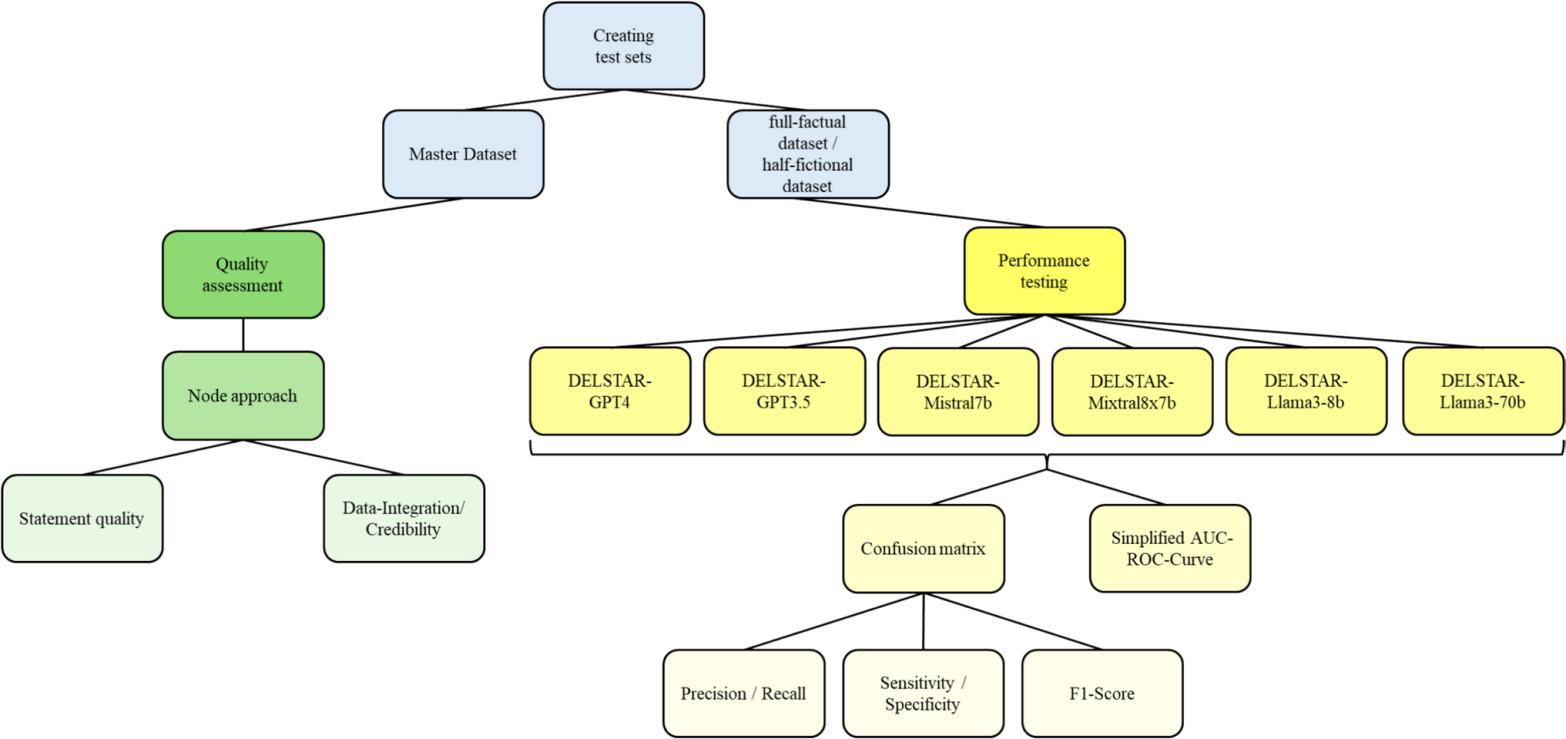
Methodology taxonomy used for quality assessment and performance testing

The system accesses offline knowledge databases and live online metadata to detect patterns between medication and delirium symptoms. The implications are that the offline model is under full control and ownership of the researcher, and live data-sources can be hand-picked and subscribed to according to need. Offline models with online source selections imply a higher level of control. A document-based dataset was translated into text embeddings and stored as vectors in a vector database. Retrieval Augmented Generation (RAG) was used to retrieve relevant data chunks to inform DELSTAR’s responses (refer to Supplementary Material 1). Bias in the dataset were controlled for by the use of systematic review in the DELSTAR database embedding and expert consultation for the selection of performance-testing questions. Figure 1 illustrates the evaluation process, with further details in subsequent sections.

### Generation of testsets

In the initial phase, three datasets—Master, full-factual, and half-fictional—were created, excluding any data used in chatbot development to ensure reliable test results. These datasets are outlined in Table 1 and described below. The Master Dataset (MD) includes all medications and related information linked to delirium, identified through three SLRs registered with PROSPERO [17, 20, 24], following Joanna Briggs Institute (JBI) and PRISMA guidelines. Identified drugs were categorised by the Anatomical Therapeutic Chemical (ATC) code classification (2023) from Bundesinstitut für Arzneimittel und Medizinprodukte (BfArM), Germany [32]. Two balanced binary datasets, each containing 50% of a shuffled MD, were created. The half-fictional dataset (D2) features fictitious drug names generated by a Mixtral 8 × 7b chatbot (temperature setting = 1). The full-factual dataset (D3) incorporates non-delirium drugs, with medication classes in the ATC code but absent from the MD classified as non-delirium-associated for testing [32]. A few-shot approach was implemented for testing, resulting in 286 drugs per dataset [33]. The data collection period spanned from 03.04.2024 to 16.07.2024.

**Table 1:** Comparison of the data sets used to test DELSTAR based on their size and composition

Name	Master dataset (MD)	Half-fictional dataset (D2)	Full-factual dataset (D3)
Number of contained drugs	290	286	286
Composition of the dataset	100% of drugs identified as delirium-associated during three state-of-the-art SLRs	50% of drugs in the Master dataset (associated with delirium); 50% of fictional drug names (not associated with delirium)	50% of drugs in the Master dataset (associated with delirium); 50% of existing drugs (not associated with delirium)

### Quality assessment

Quality testing was conducted using the MD (Sect. “Website and journal citation analysis”) with a node approach (NA) based on a simplified version of the Tree-of-Thought (ToT) strategy [34, 35]. This approach, validated to improve query success, is used when early decision-making or strategic foresight is required. In this simplified version, human experts, rather than a ToT controller AI, handled backtracking. In DELSTAR’s context, NA provided multiple response options, allowing users to explore further details and request evidence. Testing followed a structured framework (Table 2), with drug responses collated and analysed regarding their statement quality and data credibility.

**Table 2:** Framework structure encountered during chatbot search process: a search query is deemed successful only when all predefined questions in the frame have been answered according to the specifications

Initial node instruction (IN)	[NODE 0] Which medications cause delirium?
Expansion node instruction 1 (EX1)	Expand [NODE X] X and focus on specific generics. Produce new nodes for each drug
Expansion node instruction 2 (EX2)	Expand [NODE Y] Y and focus on delirium-specific information. Produce new nodes for each piece of information
Evidence node instruction (EN)	Evidence delirium-focused [NODE Y] Y and focus on the following labels: (a) Side effects, (b) Interactions, and (c) Alternatives

IN: If an error occurs, classify the error and start a new chat; EX 1 /2, EN: If an insufficient answer occurs, repeat instruction until successful

### Statement quality

Comparison of the results obtained for specific drugs using the node-based approach (NA) with the information in the Master Dataset performed by two independent expert pharmacists (KTS /IT; JS) The outcome was assigned to one of the categories (A) more specific, (B) less specific, (C) additional or (D) general. Disagreements were resolved. Results were interpreted and plotted using simple non-parametric statistics (Kruskal–Wallis-Test (*p* < 0.05)).

### Data integration and credibility

Assessment of whether DELSTAR integrates the appropriate selection of data sources and available Application Programming Interfaces (APIs). Sources cited by DELSTAR were documented and evaluated in terms of their scientific merit and quality.

### Performance testing

DELSTAR was reimplemented and exposed to external tools (online/offline foundation models) via an OpenAI-compatible API using the DELSTAR design (Sect. "Statement quality analysis"). The selection of offline open models was driven by the popularity and accessibility of models at the time of analysis, as determined by consultation with AI science community members. Lack of project funding permitted only a single closed model for use as a comparator. The online closed model was considered best-in-class at the time of the analysis. The applied test strategy allowed a well-informed assessment of chatbot performances/stability and was a foundation for implementing enhancements in DELSTAR. A simple yes–no question was automatically posed for each drug in D2 and D3:

“Does DRUG X cause delirium?”

Responses were automatically recorded, categorised as yes or no, and classified as true positives (TP), false positives (FP), true negatives (TN), or false negatives (FN) (Sect. "Statement quality analysis"). This process was applied to 6 DELSTAR-foundation model combinations with varying parameters (Fig. 1). DELSTAR(V5) was utilised for all models except Llama3, which required a Llama-specific prompt. Confusion matrices were generated, and performance metrics (recall, precision, sensitivity, specificity, F1-Score, AUC) were calculated according to best practice data mining methodology [13, 36–39]. Stability was compared using a simplified AUC-ROC based on the intersection of the calculated TPR and FPR, as no thresholds were specified.

## Results

Sections “Statement quality analysis” to “Website and journal citation analysis” serve as the foundation for the subsequent data evaluation. Since no data was collected in these sections, the results presented start with a quality assessment. As improvements were implemented in DELSTAR throughout the testing phase, the outcomes are presented chronologically. The datasets generated and analysed during this study are available from the corresponding author upon reasonable request.

### Statement quality analysis

Twenty performed NA runs were compared with the information in the MD (derived from SLRs), resulting in the final evaluation presented in Table 3. Results indicate that DELSTAR generated higher quality statements than MD in 50.0–75.0% (n = 20) cases, depending on the category. Notably, DELSTAR provided more detailed information in 75.0% (n = 20) of “Overall Content” evaluations. Conversely, the MD was rated as more specific only once within this category. While DELSTAR was categorised as less specific in 7 out of 80 categorisation options (8.8% (n = 80)) (most frequently within “Interactions”), it offered general or additional information in up to 20.0% (n = 80) of cases. The most favourable results were observed within the “Therapeutic Alternatives” category. DELSTAR generally outperformed the MD, demonstrating superior performance in most subcategories, particularly “Therapeutic Alternatives.” Notably, one instance of incorrect information generation (hallucination) was observed. A performed Kruskal–Wallis test (α = 0.05) indicated a significant difference (*p* = 0.00469) between the 5 groups suggesting varying quality level distribution.

**Table 3:** Categorisation of Responses Obtained During the DELSTAR V3 Quality Test Concerning Their Statement Quality: Two independent experts conducted a comparative analysis of the responses in the categories of side effects, interactions, therapeutic alternatives, and overall content, using the Master Dataset as the benchmark

RunID	Drug name	Side effect	Interactions	Therapeutical alternatives	Overall content
22	Fentanyl	A	B	C	A
23	Risperidone	B	B	D	D
24	Solifenacin	D	D	D	D
26	Oxycodone	B	B	D	D
27	Dexamethasone	D	D	A	D
30	Quetiapine	C	A	D	A
31	Solifenacin	A	C	B	A
32	Haloperidol	C	C	D	C
33	Trospium	A	D	D	D
34	Diazepam	A	A	D	D
35	Benztropine	D	D	B	D
36	Ropinirole	D	D	D	D
37	Hydroxyzine	B	D	D	D
39	Oxybutynin	D	D	E	D
43	Quetiapine	B	D	D	D
45	Midazolam	D	D	D	D
46	Morphine	D	C	B	B
48	Prednisone	D	A	D	D
51	Tapentadol	D	D	D	D
53	Lorazepam	D	D	D	D

The responses were classified according to their quality in A–E. The criteria used by the independent experts were guided by their professional competencies as qualified pharmacists

Data key: A = General information both in the dataset and chatbot; B = Additional information to the dataset; C = less specific than the information contained in the datatset; D = more specific than the information contained in the datatset; E = information received by the chatbot contains errors

### Citation analysis in NA runs

Analysis of 20 NA Runs revealed 94 integrated citations, predominantly supporting “Side Effects” (28.7%; n = 94) and “Interactions” (22.3%; n = 94). Citation coverage was lower for “Therapeutic Alternatives” (20.2%; n = 94) and “Specific Question” (28.7%; n = 94. While various APIs were available, only api.semanticscholar.org was explicitly utilised (n = 2), with 3 instances of access failure. Thirty-five types of sources could be identified. Website citations (x = 19; n = 35) were most prevalent, followed by journals (x = 10; n = 35) and DelstarDB databases (x = 2; n = 35). Four citations (n = 35) lacked traceability due to inaccuracies, paywalls, or missing publication links.

### Website and journal citation analysis

Website quality was assessed using criteria including authorship, review process, publication date, and update status. Website citations (n = 19) frequently lacked complete attribution, with only 79.0% (n = 19) mentioning an author or review process. Publication or update dates were present in 79.0% (n = 19) of cases, while source transparency (citations or links) was observed in 68.4% (n = 19), suggesting that DELSTAR occasionally cites websites with potentially low scientific rigour. Journal citations (n = 10) assessed using Scopus CiteScore metrics revealed an average CiteScore of 6.21, with 4 journals in Q1 (≥ 75th percentile) and 5 in Q2 (50–74th percentile). Source Normalized Impact per Paper ranged from 0.46 to 1.868, indicating a mix of above-average and below-average citation impact. While DELSTAR demonstrates the capacity to cite high-quality journals, this occurs infrequently.

### Performance testing

A series of 5 performance tests for each DELSTAR-foundation model combination and dataset were performed (refer to Fig. 1).

### Overview performance D2 and D3

**Fig. 2:**
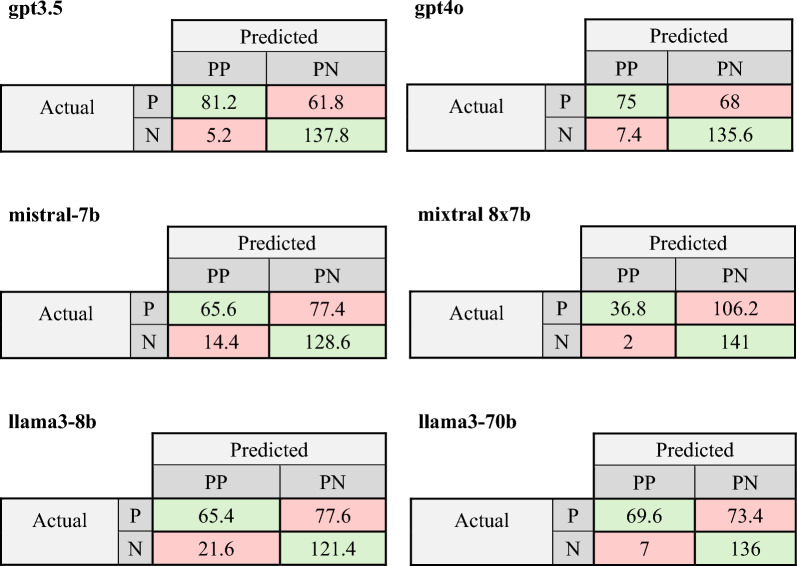
Comparison of mean DELSTAR confusion matrix test results across different platforms (GPT4o, GPT3.5, Mistral-7b, Mixtral 8×7b, Llama3-7b und Llama3-70b) for Dataset 2/3: Intersection of Predicted (predicted positives (PP), predicted negatives (PN)) and Actual Outcomes (positives (P), negatives (N))

The results showed that notably more negative predictions for all chatbot combinations were made within the binary dataset. Considering the averaged confusion matrix of Mixtral8 × 7b D3, 247.2 out of 286 cases were answered with “no” (Fig. 2). Moreover, the models yielding the highest number of “yes” responses (GPT3.5 and Llama3-70b; D3) only identified 30.4% (n = 286) of all data points as “yes”, despite the dataset being binary. In Llama3-70b, half of the positive identified results were misclassified as FP. This indicates that the chatbots tended to categorise a more significant number of medications as not associated with delirium rather than associated with delirium.

### Performance stability

All the chatbots exhibited varying degrees of performance metrics deviation in the evaluation. Notably, GPT3.5 demonstrated the least fluctuations across most of the evaluated metrics within both datasets (D2, D3), indicating a high level of consistency. Therefore, GPT-3.5 might be the most stable model for categorising medications. Mistral revealed the highest deviations in AUC values, suggesting inconsistent identification of relevant medications. Similarly, Llama3-8b exhibited substantial fluctuations, especially in D3, with a maximal deviation of 8.2% (n = 5) in the F1-Score. These substantial disparities in performance metrics suggest that Mistral and Llama3-8b may be less dependable for consistent results, highlighting the superior stability of the frontier or closed models (GPT3.5; GPT4o) by comparison. Examining the open model reveals that Llama3-70b exhibits the most stable performance for D2, while Mistral8 × 7b demonstrates the most stable performance for Dataset 3.

### Performance comparison

**Fig. 3:**
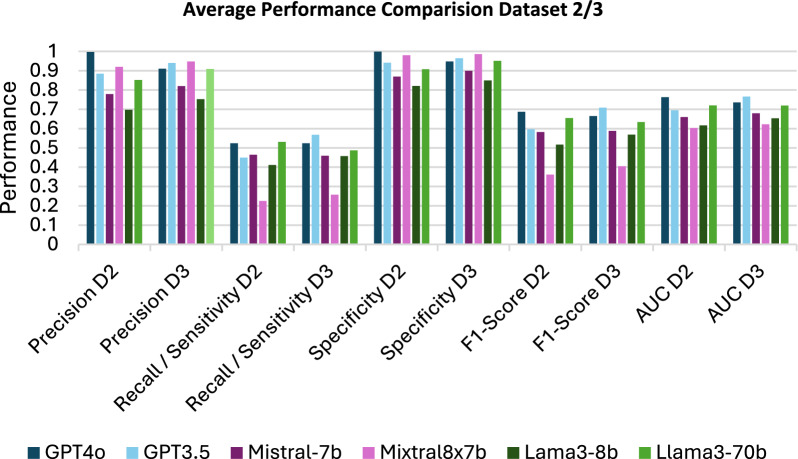
Average performance metrics results for the DELSTAR foundation model combinations using dataset 2 (D2) and dataset 3 (D3): displays the results of the calculations of the performance metrics averaged from the test runs performed

Upon reviewing the test results presented in Fig. 3, it was apparent that for D2 Llama3-70b (0.531) and GPT4o (0.524) and for D3 GPT4o (0.524) and GPT3.5 (0.568) exhibited the highest sensitivity/recall values indicating that these systems are most favourable in identifying drugs associated with delirium since the least positive instances are missed. Moreover, it revealed precision values ranging from 0.997 to 0.697, with 4 out of 6 combinations exceeding the desired threshold of 0.8 in both datasets (GPT4o, Mixtral8-7b, GPT3.5, LLama3-70b). The F1-Score, indicating the equilibrium between precision and recall, identified GPT4o (0.687) and Llama3-70b (0.655) as the most effective for D2 and GPT3.5 (0.708) and GPT4o (0,665) for D3, therefore demonstrating the most optimal performance [40]. An examination of the specificity, defined as the ability of the model to identify negative instances correctly, revealed that all chatbot combinations fall within the desired range (0.8–1.0) [41]. Consequently, the models exhibited varying degrees of capability in correctly recognising non-delirium-associated drugs, with probabilities ranging from 99.0% (n = 143) (GPT4o D2) to 82.1% (n = 143) (Llama3-8b D2). In summary, the assessment of chatbot combinations for identifying delirium-associated drugs identified GPT-4o and Llama3-70b as the top performers within D2 and GPT3.5 and GPT4o within D3. Despite achieving commendable precision values, none of the models met the desired performance threshold of 0.8–1.0 across all metrics.

## Discussion

DELSTAR has demonstrated the ability to provide accurate and comprehensive information, as indicated in prior studies [42]. This feature could be helpful in identifying complex diseases like delirium [43]. By utilising interdisciplinary approaches and expert evaluations, it became evident that DELSTAR can obtain more accurate information than traditional systematic literature searches. Given that DELSTAR semantic searching involves deeper connections between concepts then simple keyword matching alone, search performance can be augmented to include additional findings [e.g. traditional systematic literature search: Polypharmacy AND (Delirium AND medication) NOT dementia vs. Augmented literature search using DELSTAR populated with key papers and API connections: [1] RESEARCHER: Using evidence supplied, provide 5 nodes of most likely medications suspected of causing delirium inc. evidence from API search—[DELSTAR OUTPUT] [2]; RESEARCHER: Node X selected. Please elaborate -[DELSTAR OUTPUT]; [3] RESEARCHER: Provide 5 nodes of possible mechanisms of action inc. evidence from API search -[DELSTAR OUTPUT]; [4] RESEARCHER: Node Y selected. Please elaborate -[DELSTAR OUTPUT];[5] RESEARCHER: Provide 5 nodes of possible mechanisms that also cause signs of dementia inc. evidence from API search/ etc.]

The results indicate that customised LLMs have the potential to assist clinical pharmacists in synthesising scientific data from the literature, thereby enhancing access to high-quality resources [27]. Since clinical decision support systems can reduce medication errors, further refinements and research on DM seem promising [43, 44]. However, the accuracy of the systems largely depends on the quality of training data, and errors like hallucinations illustrate the importance of oversight by clinical pharmacists when using pharmaceutical/medical AI models [27]. While LLMs like DELSTAR can potentially enhance healthcare, misinformation poses significant risks to patient safety, particularly in drug safety, where incorrect information could lead to harm [45, 46]. In clinical pharmacy, relying on high-quality and credible sources is essential to ensure the safety of the medication process [27, 47]. The performed audit of the cited scientific sources regarding comprehensiveness and information quality revealed citation frequency and quality variations. Therefore, improving citation frequency and quality is necessary to demonstrate these associations effectively [48]. To enhance data quality, we implemented private databases (Delstar_DB1 and DB2). However, similar to OpenAI models, the data was not updated in real time [49]. We integrated APIs like semanticscholar.org to assess the latest research findings. Due to OpenAI’s confidentiality restrictions, this was only partially successful [12]. Consequently, a constantly updated offline model should be preferred for clinical pharmacy applications to reduce security concerns and ensure high-quality data [27]. Llama3-70b, capable of processing metadata-rich PDFs, may provide better performance in handling large-quality datasets offline [50]. Additionally, a tool like Searxng could help integrate scientific metadata into queries for local database searches, making it a valuable add-on for applications in clinical pharmacy [51]. The evolution of LLM reasoning topologies continues. A ‘Graph of thoughts’ (GoT) method has since evolved from ToT whereby feedback loops applied to prior nodes are refined into new nodes[52]. The authors note the benefits of this extension and plan to include this feature in further iterations of DELSTAR.

Performance stability is critical in medical and pharmaceutical applications to ensure consistent outputs. DELSTAR’s performance tests showed that larger models, such as GPT3.5 and Llama3-70b, exhibited the highest strength. In contrast, models with fewer parameters displayed more variability, particularly when processing datasets containing fictional drug names [49, 53]. As clinical workloads increase the likelihood of errors, more robust systems are needed to minimise the errors resulting from system instability [44]. The comparison of the performance parameters revealed a tendency to classify more medications as non-delirium-associated, which was reflected in low recall/sensitivity values. Given the health risks of missed instances, it is crucial to increase recall, for example, through internal classification benchmarks [54, 55]. In performance studies, other medical AI models have demonstrated significantly higher recall/sensitivity values, ranging from 0.800 to 0.997 [13]. It is important to note that precision and recall are interdependent; adjusting the classification threshold to increase recall may lead to a marked reduction in system precision. Therefore, ensemble methods—such as bagging, boosting, or stacking—could simultaneously enhance parameters and overall model performance [55]. Regarding performance metrics, GPT4o excelled in D2 (F1-Score: 0.687), while GPT3.5 led in D3 (F1-Score: 0.708). However, none of the models achieved the ideal threshold of 0.8, highlighting the need for fine-tuning. For offline use, Llama3-70b’s stability and performance make it the preferred model for clinical pharmacy applications in our test. Further development is needed prior to carrying out feasibility testing in the clinical setting. In addition, the ethical implications of using AI models like DELSTAR in clinical practice must be explored. While the model’s training data is not based on individual patient information it’s use in supporting patient medication therapy decisions in clinical practice needs detailed consideration. The EU AI Act and the AI Liability Directive were released in August 2024 and will guide the discussion [56, 57].

### Strengths and limitations

This simplified ToT design allows researchers to efficiently explore semantic information while controlling various variables, leading to advancements in AI-assisted clinical tools. DELSTAR significantly reduces the time needed for manual literature searches by providing rapid access to synthesised data on medications and complex diseases like delirium. While no comprehensive evaluation methods exist for LLM in clinical pharmacy, DELSTAR emphasises the need for such frameworks to improve evaluation practices for domain-specific LLM. However, this study has several methodological limitations. It was designed to illustrate system strengths and weaknesses rather than provide full validation through real-world use cases. The testing employed small, balanced datasets (< 300 cases), which may not accurately represent real-world scenarios where data balance varies. Additionally, sequential testing and continuous system development, including different OpenAI models, impacted the analyses. None of the systems fully reflects the latest research developments, as private databases need regular updates, and specific data cutoff dates constrain OpenAI’s GPT models.

### Further research

To optimise DELSTAR for clinical pharmacy practice, addressing its identified weaknesses and incorporating high-quality, evidence-based data in a secure, offline environment is essential. This approach will ensure the generation of accurate and reliable information, which is critical for safe medication management. Comprehensive testing in real-world clinical scenarios should be performed, allowing targeted refinements. Furthermore, a thorough review of the legal and regulatory framework for evaluating and approving AI tools in clinical pharmacy is necessary to guarantee their safety, reliability, and alignment with professional standards.

## Conclusion

This interdisciplinary evaluation offers valuable insights into the performance and quality of DELSTAR, a chatbot system designed to enhance medication safety in delirium. It demonstrated strengths in retrieving information on medication-associated delirium, showcasing its potential as a valuable resource for identifying and managing high-risk conditions. However, the evaluation also identified areas for improvement, particularly in ensuring the use of high-quality sources and enhancing performance metrics. With the rapid advancement of AI technologies, clinical pharmacists are encountering new opportunities and responsibilities. One key responsibility involves evaluating and monitoring AI models to identify hallucinations, prevent errors, and ensure patient safety. By addressing these challenges, tools like DELSTAR can be valuable in optimising the efficiency of clinical workflows and support evidence-based decision-making in clinical pharmacy practice. Further development and feasibility testing using real-world clinical data is needed.

## Supplementary Information

Below is the link to the electronic supplementary material.

Supplementary file1 (DOCX 23 KB)
