# Supplementary material for: Evaluating a customised large language model (DELSTAR) and its ability to address medication-related questions associated with delirium: a quantitative exploratory study
Source: Int J Clin Pharm. 2025 Apr 10;47(4):1053–63. doi: 10.1007/s11096-025-01900-8 (PMC12335389; doi:10.1007/s11096-025-01900-8)
Supplement: Supplementary file 1 — Supplementary file1 (DOCX 23 KB) [file 11096_2025_1900_MOESM1_ESM.docx]

**Supplementary Material**

*Dataset Processing*

In the context of the RAG dataset, a total of 193 PDF documents were initially converted from individual files into two collective monolithic PDF documents. Subsequently, these were transformed into two singular monolithic text files utilising a bulk operation within Adobe Acrobat Pro. During the importation process, the text was segmented into chunks of 1500 tokens, incorporating an overlap of 20 tokens between consecutive chunks. These segments were then processed using the nomic-embed-text embedding model, resulting in vectorised embeddings that were subsequently stored in a pickle-formatted file, thereby establishing the vector database.

*Retrieval Approach*

Regarding the retrieval mechanism implemented for obtaining relevant chunks to be processed by the language model, empirical testing with BM25-like models was deferred. Traditional sparse methodologies, such as BM25, predominantly rely on lexical scoring mechanisms, which essentially involve keyword matching. While these baseline methods tend to perform adequately on standard hardware, they often lack robust semantic comprehension. Within the Delstar pipeline, enhanced computational capacity facilitated the adoption of semantic retrieval tools derived from the llama_index Python library, specifically leveraging the llama_index embeddings. An auxiliary reranker post-processing step was integrated to refine the informational retrieval based on semantic context. The semantic reranker employed, FlagEmbeddingReranker, utilizes an additional model (BAAI/bge-reranker-base) that is designed to fine-tune embeddings to assess and reorder documents in accordance with their semantic relevance to the query posed.

It is important to note that FlagEmbeddingReranker is computationally intensive, which accounts for the several seconds required to search its database when responding to queries. An exploration of an alternative reranking model, ColBERT, was also conducted; however, while it yielded comparable results, it resulted in longer processing times, leading to its discontinuation. Unfortunately, timing data from the A/B testing iterations conducted was not preserved.

*Semantic Scholar Integration*

Furthermore, it is noteworthy that the use of the Semantic Scholar API was not activated in the DELSTAR NoNode Binary API version that was utilized for generating the resultant dataset. However, this feature has been incorporated in the DELSTAR Node version through the implementation of a locally-operated searxNG engine. When activated, this functionality serves to augment the RAG retrieval process by adding additional chunks sourced from text derived from the Semantic Scholar API rather than from the existing PDF vector database.
